# Correlation and predictive ability of sensory characteristics and social interaction in children with autism spectrum disorder

**DOI:** 10.3389/fpsyt.2023.1056051

**Published:** 2023-04-06

**Authors:** Jinhe Zhai, Xiaoxue Li, Yong Zhou, Lili Fan, Wei Xia, Xiaomin Wang, Yutong Li, Meiru Hou, Jia Wang, Lijie Wu

**Affiliations:** ^1^School of Public Health, Harbin Medical University, Harbin, China; ^2^Heilongjiang Provincial Center for Disease Control and Prevention, Harbin, China

**Keywords:** autism, sensory, social, core symptom, SSP, clinical scale

## Abstract

**Background:**

Individuals with autism spectrum disorder (ASD) often have different social characteristics and particular sensory processing patterns, and these sensory behaviors may affect their social functioning. The objective of our study is to investigate the sensory profiles of children with ASD and their association with social behavior. Specifically, we aim to identify the predictive role of sensory processing in social functioning.

**Methods:**

The Short Sensory Profile (SSP) was utilized to analyze sensory differences between ASD children and their peers. The Social Responsiveness Scale (SRS) and other clinical scales were employed to assess the social functioning of children with ASD. Additionally, the predictive ability of sensory perception on social performance was discussed using random forest and support vector machine (SVM) models.

**Results:**

The SSP scores of ASD children were lower than those of the control group, and there was a significant negative correlation between SSP scores and clinical scale scores (*P* < 0.05). The random forest and SVM models, using all the features, showed higher sensitivity, while the random forest model with 7-feature factors had the highest specificity. The area under the receiver operating characteristic (ROC) curve (AUC) for all the models was higher than 0.8.

**Conclusion:**

Autistic children in our study have different patterns of sensory processing than their peers, which are significantly related to their patterns of social functioning. Sensory features can serve as a good predictor of social functioning in individuals with ASD.

## Introduction

Autism spectrum disorder (ASD) is a complex neurodevelopmental disorder characterized by challenges in social communication and interaction and intense behavior or interests (1). In recent years, the global incidence of ASD has increased rapidly. According to the Centers for Disease Control and Prevention (CDC) in the United States, the prevalence of ASD has risen to 1 in 44 individuals (2). The global prevalence has reached 1−2% (3). A multicenter survey of children aged 6−12 years in China found that the prevalence of ASD was approximately 0.70% (4). The increasing prevalence has necessitated that we understand the impact of ASD.

In the past, researchers have primarily focused on social and stereotyped behaviors when studying ASD, but they have gradually discovered that many people with ASD also experience differences in sensory perception. In fact, as early as the 1940s, Kanner (5) had already described these symptoms in individuals with ASD. More recently, the fifth edition of the Diagnostic and Statistical Manual of Mental Disorders (DSM-5) has included sensory patterns as a diagnostic criterion for ASD, increasing the attention given to sensory features in ASD research. Studies have found that particular sensory patterns occur in ASD of all ages, with intense sensory experiences reported in as many as 60 to 90% of individuals with ASD (6).

Sensory processing is the brain’s way of recognizing and organizing external stimuli to obtain effective information from them (7). Living organisms are constantly exposed to a diverse array of information in their environment and rely on their sensory system to process information from various sources, such as sounds, visual stimuli, tactile sensations, and smells. For social creatures like humans, the environment is also rich in social information that needs to be perceived and understood. In a study by Morgan, it was suggested that the brain’s sensory and perceptual system evaluates and integrates social information and situational factors to generate appropriate social behaviors in response to social-emotional cues (8).

Individuals with ASD often experience intense sensory processing, which makes it difficult for them to adapt to the changing sensory environment. It is believed that sensory processing and socialization may originate from the same underlying mechanisms and that they may influence each other during childhood development. These sensory experiences have negative effects on attention and language development and may predict social withdrawal (9–11). Communication involves multiple social cues that are often combined, requiring individuals to process multiple sensory modalities simultaneously. However, existing research often focuses on a single sensory modality, and there is a lack of comprehensive evaluation of all sensory modalities in ASD and their relationship with social interaction. Therefore, investigating the relationship between multiple sensory modalities and social interaction could provide a new direction for ASD intervention and is the primary aim of this study.

Autism is mainly treated through behavior training as there is currently no drug treatment available. The effectiveness of intervention is largely dependent on the timing of its implementation: therefore, early identification of symptoms is crucial for a better prognosis. There is evidence that differential sensory behavior manifests earlier, which highlights the potential of exploring the predictive value of sensory features in estimating social levels. This not only reveals the relationship between sensory and social features but also enhances early diagnosis of autism. To this end, this study employs machine learning (ML) algorithms, such as neural networks, decision trees, rule-based classifiers, and support vector machines, which are automated data processing tools that can build accurate prediction models based on research datasets (12). The use of machine learning algorithms in autism research was initially introduced to address the challenge of diagnosing this complex disorder. In Paolo et al. (13) applied a support vector machine model and found that dyskinesia can be used as a clinical symptom to facilitate early recognition of ASD. Subsequent research has increasingly focused on the potential applications of machine learning in the field of ASD. With a deeper understanding of autism, researchers have explored a wider range of markers beyond early behavioral presentations, including neuroimaging, molecular biology, and genetic predictions, which can be combined with machine learning to provide new avenues for ASD research (14–16). For example, Alivar et al. (17) utilized support vector machines and artificial neural network classification models to examine the impact of common sleep problems in ASD on daily behavior. Despite numerous successes in incorporating machine learning into ASD research, few studies have focused on individual typical symptoms, such as social interaction.

To sum up, the present study aims to achieve two objectives: (a) investigating whether children with ASD have a higher frequency of sensory reactivity differences than their peers; and (b) examining the correlation between sensory processing, social function, and clinical diagnosis in children with ASD, as well as the predictive power of sensory features for social ability. This research is expected to provide clinical physicians with auxiliary tools for diagnosis and increase the enthusiasm of rehabilitation therapists in formulating intervention strategies that emerge from knowing a person’s sensory patterns.

## Materials and methods

### Participants

Participants for the ASD group were recruited from the Research Center for Children’s Developmental Behavior of Harbin Medical University. Criteria for inclusion in the ASD group required a diagnosis of ASD by a psychiatrist or clinical psychologist using the DSM-5 diagnostic criteria. Participants for the control group were recruited from local kindergartens and primary schools in Harbin city. Individuals with comorbid mental development disorders, definite head trauma, nervous system diseases, major somatic diseases, or a history of epileptic seizures were excluded.

The study included a total of 489 participants divided into two groups: the ASD group, consisting of 266 individuals with autism (219 boys and 47 girls), and the control group, consisting of 223 children (170 boys and 53 girls). The children in the ASD group had a mean age of 5.31 ± 0.102 years, while those in the control group had a mean age of 5.32 ± 0.115 years. There were no significant differences between the two groups in terms of age or gender.

### The Short Sensory Profile

The Short Sensory Profile (SSP) is a questionnaire completed by parents to assess children’s responses to sensory stimuli. It is commonly used to identify sensory behaviors in children with ASD between the ages of 3 and 10 years. The questionnaire consists of 38 items across seven sensory domains, each describing various sensory-related behaviors. The frequency of behaviors is scored on a scale of 1 to 5, with lower scores indicating a greater possibility of sensory differences from expected patterns. Based on the score range, the testee’s sensory profile can be categorized into three grades (expected patterns = 0, possible difference = 1, or obvious difference = 2) (18) (The specific classification criteria are shown in Supplementary Table 2). The grades of possible difference and obvious difference are regarded as unexpected patterns.

### The Social Responsiveness Scale

The Social Responsiveness Scale (SRS) is used as an aid in the clinical diagnosis of ASD children between the ages of 4 and 18 years. The scale consists of 65 items divided into 5 sub-scales, each describing the social situations children may encounter in their daily lives, including behaviors related to social communication, communication skills, and repetitive and rigid behaviors associated with autism. A higher score on the scale indicates a greater degree of social difference (19).

### Autism diagnostic interview-revised

The Autism Diagnostic Interview–Revised (ADI-R) is a standardized and structured diagnostic tool based on parent interviews and is widely recognized as the gold standard in autism assessment. The scale consists of four domains: social interaction, communication, repetitive behaviors, and early developmental history. Higher scores on the ADI-R indicate greater impairment in the corresponding domain (20).

### Autism diagnostic schedule

The Autism Diagnostic Observation Schedule (ADOS) is a standardized and semi-structured diagnostic tool that is widely recognized as a gold standard for ASD diagnosis. Specifically trained evaluators interact with children in a standardized manner, observing their behavior for signs of restricted and repetitive interests and behaviors during play and assessing their social communication, play, and imagination for the purpose of assisting in the diagnosis of ASD (21).

The questionnaire and scales used in this study were administered in Chinese. The authorized Chinese versions of these instruments were used to assess the participants’ autism symptoms and related behaviors.

## Data preprocessing of model construction

Basic participant information and SSP data were collected as key variables, including age, sex, and eight sensory dimensions of the SSP. Participants with ASD were categorized for their social responsiveness based on their SRS raw scores, with a cut-off of 60. Children who score above 60 are more likely to be considered as having autism. A two-variable prediction model was constructed using SRS scores (0 for expected patterns, 1 for unexpected patterns) as the output variable.

### Random forest model

Random forest is a type of ensemble learning method that uses decision trees as the base model, specifically, classification and regression trees (CART) (22). CART uses the Gini index as the evaluation criterion, which is defined as Gini = 1-Σ(P(i)*P(i)), where P(i) represents the proportion of type I samples in the data set at the current node. The ntree parameter is tested and adjusted during the model-building process. The Gini index is used to calculate the heterogeneity of the observed values at nodes in the classification tree caused by each variable, which allows for the comparison of variable importance. The model is re-constructed for each method by retaining the feature factors with the highest importance ranking after calculating the relationship between the error rate and the number of features through cross-validation.

### Support vector machine model

Support vector machine (SVM) is a classification model that is based on the principle of structural risk minimization and VC dimension theory. This model seeks the best balance between learning ability and accuracy to obtain the best generalization ability (23). In this study, the Gaussian kernel function that demonstrated the best performance was used as the kernel function for the SVM model. Initially, all feature factors were inputted into the SVM model for training. Subsequently, based on the variable importance from the random forest model, the feature factors with high importance were selected to reduce the feature factor dimension. The selected feature factors were then inputted into the SVM model for further training.

### Model construction and verification

To mitigate structural risks in the model construction process and evaluate the generalization ability of the prediction model, we adopted the K-fold cross-validation method. In each fold, 9/10 of random samples were used for model training, and the remaining 1/10 were used for model validation. By separating the data into training and validation sets, the model’s effectiveness could be better assessed. We performed a 10% discount cross-validation five times. The model’s prediction performance was evaluated based on its sensitivity and specificity in predicting the test set and the AUC under the ROC curve.

## Statistical analysis

The original data were analyzed using SPSS version 26.0. The Shapiro-Wilk (S-W) method was used to test the normality of scores for each scale. The Chi-square test was used to determine whether there was a difference in gender between the ASD group and the control group. The Mann–Whitney U test was used to determine if there were differences in age and SSP scores between the two groups. The Chi-square test was used to analyze differences between the ASD and control groups in SSP score grades. In addition, Spearman rank correlation was used to analyze the correlation between SSP scores and clinical scales in the ASD group. The random forest and support vector machine models were constructed and verified using R version 3.5.3.

## Results

### Analysis of differences in sensory features between groups

The S-W test showed that the SSP score data of the two groups did not obey the normal distribution. According to the Mann–Whitney test, the scores of the ASD group in seven sub-fields and the total scores of multiple senses were significantly lower than those of the comparison group. The χ^2^ test showed that the ratio of unexpected patterns in the ASD group was higher than that of the TD group (*P* < 0.05) (Table 1).

**TABLE 1:** Comparison of SSP score characteristics between ASD group and healthy control [Mid (P25, P75)/*n* (%)].

		ASD (*n* = 266)	TD (*n* = 223)	Z/χ^2^	*P*
Tactile	Mid (P25, P75)	33 (30, 35)	34 (31, 35)	−2.73	0.005
	Expected pattern (0)	213 (80.4%)	197 (88.3%)	5.719	0.017
	Unexpected patterns (1 + 2)	52 (19.6%)	26 (11.7%)		
Gustation/olfaction	Mid (P25, P75)	18 (15,20)	19 (17, 20)	−4.361	<0.001
	Expected pattern (0)	199 (74.8%)	202 (90.6%)	20.445	<0.001
	Unexpected patterns (1 + 2)	67 (25.2%)	21 (9.4%)		
Movement sensitivity	Mid (P25, P75)	13 (11, 15)	14 (12, 15)	−3.168	0.002
	Expected pattern (0)	157 (59.2%)	152 (68.5%)	4.431	0.035
	Unexpected patterns (1 + 2)	108 (40.8%)	70 (31.5%)		
Hypo-sensitivity/Sensory seeking	Mid (P25, P75)	28 (26, 31)	31 (27, 33)	−5.087	<0.001
	Expected pattern (0)	70 (68.3%)	176 (79.6%)	7.942	0.005
	Unexpected patterns (1 + 2)	84 (31.7%)	45 (20.4%)		
Auditory filtering	Mid (P25, P75)	19 (16, 22)	24 (21, 28)	−10.995	<0.001
	Expected pattern (0)	59(22.2%)	144(65.2%)	91.723	<0.001
	Unexpected patterns (1 + 2)	207 (77.8%)	77 (34.8%)		
Low strength	Mid (P25, P75)	22 (18, 27)	28.5 (25, 30)	−10.452	<0.001
	Expected pattern (0)	90 (33.8%)	165 (74.3%)	79.515	<0.001
	Unexpected patterns (1 + 2)	176 (66.2%)	57 (25.7%)		
Vision/Auditory	Mid (P25, P75)	20 (17, 22)	21 (19, 23.25)	−3.426	0.001
	Expected pattern (0)	180 (67.7%)	170 (76.9%)	5.113	0.024
	Unexpected patterns (1 + 2)	86 (32.3%))	51 (23.1%)		
Total score of multiple senses	Mid (P25, P75)	152 (138, 164)	167 (155, 178)	−9.534	<0.001
	Expected pattern (0)	118 (44.9%)	171 (78.8%)	57.150	<0.001
	Unexpected patterns (1 + 2)	145 (55.1%)	46 (21.2%)		

ASD, autism spectrum disorder; TD, typically developing control; 0 means that the SSP questionnaire score is within the expect pattern, 1 means that the score is within the possible difference, and 2 means that the score is obviously difference; % The percentage of SSP score in the corresponding level to the total number of people.

According to the nature of each topic in the SSP questionnaire, we divided all items into three modes: hyper-sensitivity, hyposensitivity, and seeking, and described the number of ASD people with these three patterns, respectively. The number of people with only one of the patterns was 95 [hypersensitivity = 84 (31.6%), hyposensitivity = 7 (2.63%), sensory seeking = 4 (1.5%)]. In addition, 69 people had two types of sensory reactivity differences at the same time, and 59 people showed all three types of patterns at the same time (Supplementary Table 1).

### Correlation analysis between SSP score and behavior scale score in the ASD group

Spearman correlation analysis showed that the SSP gustatory/olfactory and motor sensitivity scores were negatively correlated with ADI-R social function scores, tactile scores were negatively correlated with non-verbal communication scores, but the correlation with the score of verbal communication was not statistically significant; the gustation/olfactory score was positively correlated with the ADOS communication score; and the auditory filtering score was negatively correlated with the ADOS social interaction score. Except for the correlation between motor sensitivity and all dimensions of SRS, the scores of other sensory dimensions of SSP were negatively correlated with certain domains of SRS (*P* < 0.05). The correlation between the SSP score and other dimensions of each scale score is not statistically significant (*P* > 0.05) (Table 2).

**TABLE 2:** Spearman correlation analysis between SSP score and SRS/ADIR/ADOS score(r).

	SRS (*n* = 113)						ADI-R (*n* = 90)		ADOS (*n* = 94)	
	Social perception	Social cognation	Social communication	Social motivation	Autism behavior	Total score	Non-verbal communication	Social function	Communication	Social interaction
Tactile	−0.186	−0.226*	−0.281**	−0.221*	−0.110	−0.276**	−0.267*	−0.161	0.134	−0.179
Gustation/olfaction	−0.228*	−0.177	−0.189*	−0.184	−0.264**	−0.229*	−0.153	−0.207*	0.229*	0.044
Movement sensitivity	−0.120	−0.109	−0.109	−0.178	−0.026	−0.141	−0.134	−0.212*	0.100	−0.073
Hypo-sensitivity/sensory seeking	−0.346**	−0.409**	−0.435**	−0.391**	−0.474**	−0.528**	−0.154	−0.154	0.048	−0.182
Auditory filtering	−0.312	−0.263**	−0.444*	−0.423**	−0.274**	−0.456**	−0.138	−0.166	−0.085	−0.253*
Low strength	−0.165	−0.173	−0.217*	−0.081	−0.000	−0.189*	0.162	0.072	−0.017	−0.058
Vision/auditory	−0.189*	−0.138	−0.194*	−0.238*	−0.264**	−0.258**	−0.072	−0.188	0.075	−0.030
Total score of multiple senses	−0.379**	−0.352**	−0.449**	−0.360**	−0.345**	−0.485**	−0.112	−0.168	0.059	−0.150

*P < 0.05 and **P < 0.01. SRS, social responsiveness scale; ADI-R, autism diagnostic interview-revised; ADOS, autism diagnostic schedule.

### Data dimension reduction results of the models

The varImpPlot function in random forest is used to rank the importance of all factors, reduce the dimension of characteristic factors, select the characteristic factors that have a significant influence on the prediction results, and reconstruct the prediction model. The order of importance is hyposensitivity/sensory seeking>*multi*−*sensory*>*age*> auditory filtering>low strength>vision/auditory>*tactile*>gustation/olfactory>motor sensitivity>gender. According to the cross-validation of the relationship between error rate and characteristic factors, when the number of characteristic factors is approximately seven, the error rate is low and stable. Therefore, the first seven characteristic factors are selected to construct the random forest model and SVM model again, and the prediction results are compared with those of the ten characteristic prediction models.

### Analysis, evaluation, and comparison of random forest model and SVM model

**FIGURE 1:**
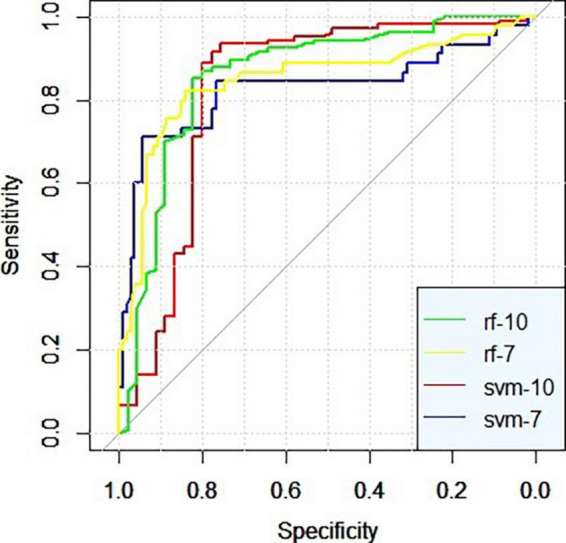
ROC curves of the models.

Both the stochastic forest model and SVM model with all feature factors have higher sensitivity, specificity, and AUC than the model with data dimension reduction. The random forest model with all characteristic factors has the highest sensitivity, up to 0.916, and the stochastic forest model with seven characteristic factors has the highest specificity, reaching 0.944. The ROCs of the four curves are close to the upper left corner, and AUC is higher than 0.8. The prediction effect of the models is good (0.8 ∼ 0.9). Among them, the SVM prediction model with all feature factors has the best AUC (Figure 1 and Table 3).

**TABLE 3:** Comparison of invalidation result of prediction models.

Model category	Sensitivity	Specificity	AUC
10-Factors random forest	0.916	0.778	0.828
7- Factors random forest	0.711	0.944	0.827
10-Factors SVM	0.850	0.822	0.859
7- Factors SVM	0.822	0.841	0.848

## Discussion

The sensory system is complex and the differences in the performance of ASD individuals can manifest in various ways, making it difficult to distinguish and evaluate sensory behavior. As a result, there have been numerous studies on sensory symptoms in ASD utilizing many types of sensory questionnaires. For instance, Baranek et al. (24) found that 69% of ASD children had sensory differences using the Sensory Experiences Questionnaire (SEQ). Rogers et al. (25), using the SSP, found that individuals with ASD were more sensitive to tactile, auditory, and taste/smell stimuli. Tavassoli et al. (26), in a similar survey using the Sensory Profile (SP), found that 31% of children with neurodevelopmental disorders, including ASD, had auditory over-responsivity and 27% had tactile over-responsivity. However, the previous studies have not clearly distinguished the differences in performance and their severity among sensory domains. The innovation of the current study lies in its use of the SSP questionnaire to analyze evaluation results in detail and address the aforementioned issues. Our findings indicate that within the ASD group, 55.1% of total scale scores were classified as unexpected patterns, suggesting that over half of children with ASD exhibit more intense sensory behaviors in general.

During our further in-depth analysis, we noted that the highest proportion of differences in sensory behavior was related to auditory filtering (77.8%). The ability to extract and process relevant information from complex sound environments is essential in daily communication. Most parents in our study reported that their ASD children had difficulty concentrating and performing activities in noisy environments, indicating that it is not easy for ASD individuals to filter out irrelevant sounds. Previous research by Groen found that individuals with autism require a higher signal-to-noise ratio than control groups to extract disyllabic words from various auditory backgrounds, as demonstrated in their performance on sounds presented in pink noise (27). Moreover, when combined with the 30% of ASD children who exhibit sensory differences in the visual and auditory domains, our findings suggest a comprehensive difference in audio-visual integration within this group. Previous research found that ASD children’s audio-visual temporal binding window is four times larger than that of typically developing children, indicating a significant deficit in audio-visual acuity (28). Another sensory dimension with a high proportion of different behavior was related to the tactile system, with 66.2% of children with ASD displaying sensory-motor problems. Specifically, 40.8% of the children in this group were found to be sensitive to movement. Previous studies have documented a range of sensorimotor disorders in ASD, including uncoordinated movements of the upper and lower body, manifesting as difficulty with gait and reaching, respectively (29–31). Of particular note in this study is the observation that individuals with ASD have difficulty with tasks requiring strong grip strength, lifting heavy objects, and maintaining balance and posture. These findings provide new evidence and insight into the sensory-motor performance of individuals with ASD. Dorothea has suggested that ineffective motor circuits may be the underlying cause of autism motor patterns (32). Although the unexpected patterns’ rates of other sensory domains in our sample of individuals with ASD were relatively low, they were still significantly higher than in comparison children and could contribute to behavioral differences in ASD. For example, their tactile sensitivity may make them uncomfortable with being approached or touched by others, while taste and smell sensitivity may lead to picky eating and anorexia, which can hinder individual development. It is clear that the sensory behavioral characteristics of ASD are varied, and a deeper understanding of them can inform targeted interventions.

In our investigation, the vast majority of individuals with ASD displayed not just one but multiple coexisting sensory reactivity differences, and they may each have their own unique effects on behavior. For instance, children with both tactile and auditory filtering difficulties may be resistant to being approached and often fail to respond to others’ calls, thus creating significant obstacles to effective communication. Furthermore, external environmental stimuli often involve a complex mix of sensory information, such as the rapid integration of auditory and visual cues in everyday conversation, which can result in speech perception deficits if not properly integrated in a timely manner (33, 34). It is clear that the presence of multiple sensory reactivity differences can exacerbate the already complex sensory issues experienced by individuals with ASD and must be taken into account when designing interventions to address these challenges. The presence of sensory responsivity in multiple dimensions makes it difficult for individuals with ASD to cope. Additionally, changes in reaction patterns play an important role in their unusual behavior, which can be classified into three categories: hyperresponsiveness, hyporesponsiveness, and sensory seeking. Our results show that among individuals with only one unusual reaction (42%), hypersensitivity was the most common (37%). However, the proportion of individuals with multiple reactions should not be ignored (57%) and is consistent with findings from Taylor’s research (35). The causes of different patterns in ASD sensory behavior have been extensively researched and may be influenced by factors such as cortical excitability, unexpected receptor function at the molecular level, and genes (36–38). However, the cross-disciplinary nature of these anomalies and the complicated reaction patterns make it difficult to distinguish ASD’s sensory behavior, which can have serious implications for their other skills. In conclusion, the detailed exploration of sensory response behavior in ASD children in current research is of great significance for treatment and symptom recognition.

Effective communication and successful social interaction require individuals to fully utilize their sensory systems in order to obtain valuable information and engage in social activities in a world full of chaotic information (39). However, particular sensory responses in individuals with ASD may contribute to the development of social disorders. The current research results show a negative correlation between SSP scores and social dimension scores on clinical scales, strongly suggesting that as sensory sensitivities become more severe, social engagement becomes more challenging. Tactile perception is particularly important for infants as it is one of the primary ways they explore the external environment and is crucial to their overall development. Tactile input, particularly friendly forms of social touch like hugs, is vital for social development from infancy onward, and gentle emotional touch can help to alleviate anxiety and improve social functioning (40). Autistic individuals often exhibit hypersensitivity to gentle touch, but reduced sensitivity to painful stimulation (41, 42), leading to difficulty in detecting social cues. The current study found that the SSP tactile score was negatively correlated with the ADI-R non-verbal communication score, which is consistent with the findings of Foss-Feig et al. (43). It is evident that tactile processing can affect various forms of communication and interaction (44). The link between tactile processing and social symptoms in individuals with ASD is also apparent, given that tactile information is critical for distinguishing the relationship between oneself and others, which is the foundation of social cognition (45). As indicated in the SRS questionnaire, autistic children may struggle to comprehend the interrelation of things, unlike their peers, which can hinder their social engagement.

Alongside touch, the impact of vision and hearing on social behavior also cannot be overlooked. “Listening” and “observing” are two essential means of communication with others. Our findings suggest that unexpected audio-visual perception is negatively associated with social functioning across three dimensions: social perception, social communication, and social motivation. Emotional recognition is a crucial aspect of social behavior, encompassing the comprehension of facial expressions and emotional rhythms (46, 47), which rely on intact audio-visual processing. Up to 80% of environmental information is transmitted to the brain through vision (48), and visual processing is linked to social behaviors such as joint attention and imitation of others (49, 50). However, most individuals with ASD find maintaining eye contact challenging and tend to focus more on local details rather than the whole, which can make it challenging to track complex and subtle social cues. ASD children often fixate on lip movements during conversations with others, rather than comprehensively observing facial expressions to discern social cues such as emotions (51). Furthermore, linguistic elements play a significant role in conveying emotions and attitudes. Due to auditory sensitivities, children with ASD may fixate on certain linguistic features (such as tone of voice or intonation) during communication, making it challenging for them to filter out socially irrelevant information from others’ speech and subsequently integrate social cues, such as linguistic expressions of emotions. Moreover, excessive sensitivity to sound may lead to children experiencing heightened fear toward particular sounds, further limiting their social development. Our assessment using the SSP has shown that ASD children often struggle to concentrate in noisy environments, underscoring the obstacles they face in auditory filtering and attention.

Anorexia, picky eating, and difficulty eating are common in children with autism, indicating a possible link to heightened olfactory and taste perception. Another key finding of this study is that these sensory differences not only affect the eating behaviors of those with ASD but may also lead to social difficulties. Through our correlation analysis, we discovered that olfactory and taste expression are also linked to the manifestation of autistic behaviors. The SRS scale defines autism behaviors as unacceptable changes in daily routines, unusual perceptual interests, and behaviors that make the child seem odd or unusual to others. These behaviors not only interfere with the emotions of individuals with ASD but also make it difficult for them to fit in socially and represent a significant obstacle to integrating into their social environment. Lahera’s research highlights the close relationship between smell perception, emotional behavior, and cognitive ability (52).

Social function is a critical factor that impacts the quality of life and prognosis of individuals with ASD. Clinical evaluations of communication ability in individuals with ASD typically involve questionnaire surveys, psychological measurements, and evaluations by appraisers during gameplay (53–55). However, these methods are often influenced by subjective factors to some extent. In recent years, with the advancement of computer technology, big data analysis methods are increasingly being employed in scientific research, and machine learning is increasingly being used in the field of ASD. Currently, machine learning algorithms are being applied in many areas of research, including the discovery of effective biomarkers for diagnosis, the classification of ASD based on various phenotypes, and the prediction of risk genes (56–58). In this study, we have innovatively used this method to determine how much sensory behavior can predict social function. Our results significantly demonstrate that both random forest and SVM models can reliably and consistently distinguish ASD social interaction, providing a new perspective for future research. The SRS scale, which serves as an output factor in our model, is widely used in clinical practice and is a necessary tool for accurately assessing ASD diagnosis and social functioning in children. The characteristic factors used in the model construction are derived from the scores of each sensory field in the SSP questionnaire, as well as demographic characteristics such as gender and age. Our findings provide evidence for predicting the possibility of ASD diagnosis and social functioning through sensory expression.

We ranked the contribution of characteristic factors to the model using the data dimension reduction method and found that the top three factors are hyposensitivity/sensory seeking, the total multi-sensory score, and age. These factors play a significant role in predicting social function. However, the importance of scores in each sensory sub-field ranked lower than the total score, suggesting that the influence of feeling on social interaction is the result of the synthesis of various senses, and the total effect is greater than the single effect. Cross-multisensory fusion’s influence on individual behavior has attracted widespread attention as a separate field, particularly for autistic people. To improve adaptive behavior, ASD interventions and treatments have been carried out using multi-sensory-based approaches (59). Electrophysiological and neuroimaging studies have established that neural circuits and brain structure play a crucial role in multisensory integration, which provides a basis for exploring the biological links between sensation and social interaction (60). The predictive effect of demographic characteristics indicates that age is the most robust factor in the model, whereas gender is the least important among the various characteristics. Currently, the diagnosis of autism lacks effective biomarkers, mainly relying on doctors’ experience in evaluating symptoms. After diagnosis, there is no specific drug for treatment, and, therefore, behavioral intervention is the only option. The results of this study emphasize that taking unusual tactile symptoms as a new perspective and paying more attention to tactile representation can not only enhance the specificity of early recognition but also improve the tactile sense as an intervention target, which is likely to alleviate ASD symptoms. These findings provide an important reminder to researchers, doctors, and even caregivers of ASD, which is that increasing awareness and sensitivity to different sensory symptoms in autistic children can greatly benefit early identification and treatment. It also emphasizes the importance of caregivers paying attention to their children’s sensory behaviors in daily life to avoid overlooking early symptoms of ASD. Furthermore, we revealed a link between sensory and social and found that sensory symptoms can reliably predict social functioning. The establishment of the autism prediction model has provided certain clinical assistance for diagnosis. As the development of sensory behavior may precede social behavior and is easier to identify, this model can predict an individual’s social level at an earlier stage, thereby increasing the sensitivity of ASD diagnosis. In addition, during the intervention process for individuals with autism, rehabilitation therapists can use this model to understand the stage-wise progress of their social functioning. All these results can help medical professionals deepen their understanding of the relationship between these two factors and provide new avenues for developing treatment strategies from a sensory perspective.

## Conclusion

This study illustrates that ASD children have a higher incidence of sensitive sensory behavior, which seems to relate to social performance, so it is important to predict social engagement based on sensory symptoms.

## Data availability statement

The data analyzed in this study is subject to the following licenses/restrictions: Due to the nature of this research, participants of this study did not agree for their data to be shared publicly, so supporting data is not available. Requests to access these datasets should be directed to JW, wangjiahyd@163.com.

## Ethics statement

The studies involving human participants were reviewed and approved by the Harbin Medical University Ethics Committee. Written informed consent to participate in this study was provided by the participants’ legal guardian/next of kin.

## Author contributions

JZ conceived the study, performed the data acquisition and analysis, and drafted and revised the manuscript. XL and YZ were involved in the writing and revision of the manuscript and contributed to the acquisition of data. LF, YL, and MH were responsible for personnel recruitment and questionnaire distribution. WX and XW conducted questionnaire recovery and quality checks. JW and LW conceived the study and helped revise the manuscript. All authors read and approved the final manuscript.
