# Supplementary material for: Correlation and predictive ability of sensory characteristics and social interaction in children with autism spectrum disorder
Source: Front Psychiatry. 2023 Apr 6;14:1056051. doi: 10.3389/fpsyt.2023.1056051 (PMC10117963; doi:10.3389/fpsyt.2023.1056051)
Supplement: Supplementary file 1 [file Table_1.docx]

**Table 1 Suppl. SSP questionnaire specific items for the three sensory response patterns**

| **Items of sensory** | |
| --- | --- |
| ***Sensory hyper-sensitivity*** | |
| **Tactile hyper-sensitivity** | |
| 1. During personal hygiene activities such as cutting hair, washing face, and trimming nails, there may be signs of resistance and crying. | |
| 2. Likes to wear long-sleeved clothes when the weather is warm or short-sleeved clothes when the weather is cold. | |
| 3. Dislikes being barefoot, especially walking on sand or grass without shoes. | |
| 4. Has a strong reaction or even aggressive behavior towards physical touch. | |
| 5. Be afraid of splashing water. | |
| 6. Be very unwilling to queue up or be close to others. | |
| **Olfactory hyper-sensitivity** | |
| 8. Refuses to eat certain foods with specific flavors or smells that are part of a normal child's diet. | |
| 9. Only eats foods with specific flavors. | |
| 10. Can only tolerate foods with specific textures or temperatures. | |
| 11. Is a picky eater, especially when it comes to food texture. | |
| **Motor sensitivity** | |
| 12. Exhibits anxiety or discomfort when both feet are off the ground. | |
| 13. Be afraid of descending from heights or has a fear of heights. | |
| 14. Dislikes activities that involve being upside down (such as doing a somersault). | |
| **Auditory fileting hyper-sensitivity** | |
| 22. Has difficulty focusing or cannot do anything when the surrounding environment is noisy. | |
| 24. Cannot concentrate on tasks when there is noise in the environment, such as from a fan or refrigerator. | |
| 25. Has difficulty completing tasks when a radio is playing. | |
| 27. Has difficulty concentrating. | |
| **Auditory hyper-sensitivity** | |
| 34. Has adverse reactions to sudden or loud noises, such as crying or hiding when hearing sounds like a vacuum cleaner, barking dogs, or a hair dryer. | |
| 35. Often covers ears with hands to avoid hearing certain sounds. | |
| **Visual hyper-sensitivity** | |
| 36. Feels uncomfortable with brightness that most people can tolerate. | |
| 37. Tends to stare at people who are walking back and forth in a room for extended periods of time. | |
| 38. Will cover or squint their eyes to avoid bright light that is acceptable to most people. | |
| ***Sensory Seeking*** | |
| 15. Likes some strange sounds or deliberately creates noise for fun. | |
| 16. Seeking various stimuli, and it has already affected daily life (such as inability to sit still, constantly making small movements, etc.). | |
| 17. Getting overly excited during sports or physical activities. | |
| 18. Enjoy touching people or objects. | |
| 20. Quickly jumping from one activity to another, to the point of disrupting games. | |
| ***Sensory hypo-sensitivity*** | |
| **Tactile hypo-sensitivity** | |
| 19. Not noticing when their own face or hands are dirty. | |
| 21. Not paying attention to clothes being twisted or bunched up on their body. | |
| **Auditory hypo-sensitivity** | |
| 23. Seemingly often not hearing what others say (such as not being able to listen or ignoring them when they speak). | |
| 26. No response when called by name, but auditory function is normal. | |
| **Sensory pattern characteristics** | **% (number/total)** |
| Expect sensory pattern | 16.2% (43/266) |
| Only sensory hyper-sensitivity | 31.6 (84/266) |
| Only sensory hypo-sensitivity | 2.6% (7/266) |
| Only sensory seeking | 1.5% (4/266) |
| Sensory hyper-sensitivity combined with hypo-sensitivity | 17.3% (46/266) |
| Sensory hyper-sensitivity combined with seeking | 7.5% (20/266) |
| Sensory hypo-sensitivity combined with seeking | 1.1% (3/266) |
| Sensory hyper-sensitivity combined with hypo-sensitivity and seeking | 22.2% (59/266) |
| only one of the patterns (95) = Only sensory hyper-sensitivity + Only sensory hypo-sensitivity + Only sensory seeking | |
| two kinds of sensory reactivity differences (69) = Sensory hyper-sensitivity combined with hypo-sensitivity + Sensory hyper-sensitivity combined with seeking + Sensory hyper-sensitivity combined with hypo-sensitivity and seeking | |
| all three kinds of patterns (59) = Sensory hyper-sensitivity combined with hypo-sensitivity and seeking | |

**Table 2 Suppl. SSP score classification standard**

| Sensory dimension | Total score | Expected patterns | Possible difference | Obvious difference |
| --- | --- | --- | --- | --- |
| Tactile sensitivity | 35 | 35-30 | 29-27 | 26-7 |
| Olfactory/taste sensitivity | 20 | 20-15 | 14-12 | 11-4 |
| Motor sensitivity | 15 | 15-13 | 12-11 | 10-3 |
| Hypo-sensitivity/Sensory seeking | 35 | 35-27 | 26-24 | 23-7 |
| Auditory filtering function | 30 | 30-23 | 22-20 | 19-6 |
| Low strength | 30 | 30-26 | 25-24 | 23-6 |
| Vision/Auditory sensitivity | 25 | 25-19 | 18-16 | 15-5 |
| Multi-sensory total score | 190 | 190-155 | 154-142 | 141-38 |
